# Sigma-1 Receptor Plays a Negative Modulation on N-type Calcium Channel

**DOI:** 10.3389/fphar.2017.00302

**Published:** 2017-05-26

**Authors:** Kang Zhang, Zhe Zhao, Liting Lan, Xiaoli Wei, Liyun Wang, Xiaoyan Liu, Haitao Yan, Jianquan Zheng

**Affiliations:** ^1^State Key Laboratory of Toxicology and Medical Countermeasures, Beijing Key Laboratory of Neuropsychopharmacology, Department of Biochemical Pharmacology, Beijing Institute of Pharmacology and ToxicologyBeijing, China; ^2^Department of Neurobiology, Beijing Institute of Basic Medical SciencesBeijing, China

**Keywords:** sigma-1 receptor, N-type Ca^2+^ channel, electrophysiology, ion channels modulation, protein–protein interaction

## Abstract

The sigma-1 receptor is a 223 amino acids molecular chaperone with a single transmembrane domain. It is resident to eukaryotic mitochondrial-associated endoplasmic reticulum and plasma membranes. By chaperone-mediated interactions with ion channels, G-protein coupled receptors and cell-signaling molecules, the sigma-1 receptor performs broad physiological and pharmacological functions. Despite sigma-1 receptors have been confirmed to regulate various types of ion channels, the relationship between the sigma-1 receptor and N-type Ca^2+^ channel is still unclear. Considering both sigma-1 receptors and N-type Ca^2+^ channels are involved in intracellular calcium homeostasis and neurotransmission, we undertake studies to explore the possible interaction between these two proteins. In the experiment, we confirmed the expression of the sigma-1 receptors and the N-type calcium channels in the cholinergic interneurons (ChIs) in rat striatum by using single-cell reverse transcription-polymerase chain reaction (scRT-PCR) and immunofluorescence staining. N-type Ca^2+^ currents recorded from ChIs in the brain slice of rat striatum was depressed when sigma-1 receptor agonists (SKF-10047 and Pre-084) were administrated. The inhibition was completely abolished by sigma-1 receptor antagonist (BD-1063). Co-expression of the sigma-1 receptors and the N-type calcium channels in Xenopus oocytes presented a decrease of N-type Ca^2+^ current amplitude with an increase of sigma-1 receptor expression. SKF-10047 could further depress N-type Ca^2+^ currents recorded from oocytes. The fluorescence resonance energy transfer (FRET) assays and co-immunoprecipitation (Co-IP) demonstrated that sigma-1 receptors and N-type Ca^2+^ channels formed a protein complex when they were co-expressed in HEK-293T (Human Embryonic Kidney -293T) cells. Our results revealed that the sigma-1 receptors played a negative modulation on N-type Ca^2+^ channels. The mechanism for the inhibition of sigma-1 receptors on N-type Ca^2+^ channels probably involved a chaperone-mediated direct interaction and agonist-induced conformational changes in the receptor-channel complexes on the cell surface.

## Introduction

Sigma receptors are originally discovered in the central nervous system of mammals in 1976 (Martin et al., 1976). Two subtypes of sigma receptors have been distinguished: sigma-1 and sigma-2 receptors, based on their different drug selectivity patterns and molecular weights (Quirion et al., 1992). Until now, sigma-2 receptors have not been cloned yet (Xu et al., 2011). On the contrary, the biological and physiological roles of the sigma-1 receptor have been examined more intensively, as it has been cloned in mice, rats and humans (Kekuda et al., 1996; Seth et al., 1998, 2001). The crystal structure of the human sigma-1 receptor is also available now (Schmidt et al., 2016). It is characterized as a 26 kDa single polypeptide containing 223 amino acids. The overall structure of the sigma-1 receptor reveals a trimeric organization. It contains only a single transmembrane domain for each promoter, and the domain includes a cupin-like β-barrel with the ligand-binding site buried at its center. Sigma-1 receptors are widely distributed in the brain and periphery organs, including lung, kidney, liver, pancreas, spleen, and adrenal gland (Hayashi and Su, 2007; Su et al., 2016). They are predominantly localized at the endoplasmic reticulum and mitochondrial-associated endoplasmic reticulum membrane (MAM, Langa et al., 2003; Marriott et al., 2012). As a molecular chaperone (Su et al., 2010), the sigma-1 receptor participates in many biological processes including nociception, cancer, stroke, memory, drug addiction, cardiac activity, and Alzheimer’s disease (Romieu et al., 2004; Renaudo et al., 2007; Tsai et al., 2009; Luty et al., 2010; Ruscher et al., 2011; Kourrich et al., 2012; David et al., 2013). Several lines of evidence reveal that sigma-1 receptors regulate a number of neurotransmitter systems, including the glutamatergic, dopaminergic, serotonergic, noradrenergic, and cholinergic systems (Cobos et al., 2008; Su et al., 2016), and many types of ion channels, including voltage-dependent Kv1.2 (Kourrich et al., 2013), Kv1.3 (Kinoshita et al., 2012), Kv1.4 (Lupardus et al., 2000; Aydar et al., 2002), voltage-gated calcium channels (Sabeti et al., 2007; Tchedre et al., 2008), Na_v_1.5 (Fontanilla et al., 2009; Johannessen et al., 2009), hERG (human Ether-à-go-go Related Gene) channel (Crottès et al., 2011) and acid-sensing ion channel (Carnally et al., 2010). It has been established that sigma-1 receptors maintain Ca^2+^ homeostasis and modulate Ca^2+^ signal through the inositol triphosphate (IP3) receptor (Hayashi and Su, 2001, 2003; Pal et al., 2008). The sigma-1 receptor has been considered as an important therapeutic target for treatment of many forms of neurodegenerative diseases in human (Guitart et al., 2004; Vidaltorres et al., 2014), even though the mechanism (Su and Hayashi, 2003) and structural basis for the regulation produced by sigma-1 receptor agonists remain poorly defined.

The N-type Ca^2+^ channel is a type of voltage-gated calcium channels (VGCCs). It mainly locates at presynaptic membrane and mediates rapid Ca^2+^ influx into the synaptic terminal that triggers synaptic vesicle exocytosis and neurotransmitter release (Llinás et al., 1981; Catterall, 2000; Momiyama and Koga, 2001). Inhibition of the N-type Ca^2+^ channels can regulate neuropsychiatric disorders in animals and humans and it has become a potential target for the treatment of certain types of pain, particularly neuropathic pain (Altier et al., 2006). It is obvious that both of sigma-1 receptors and N-type Ca^2+^ channels are involved in the same biological processes: calcium homeostasis regulation and neurotransmitter release. Sigma-1 receptor activation has been found to inhibit glutamate release from rat cortical nerve terminals by blocking N-type and P/Q-type Ca^2+^ channels (Lu et al., 2012). Despite many researches have revealed that sigma-1 receptors regulate various types of VGCCs probably by constitutive interaction (Chu and Ruoho, 2015), it is not convinced whether there is an interaction between sigma-1 receptors and N-type Ca^2+^ channels, and how the sigma-1 receptor regulates the neurotransmission.

Considering their important functions in human physiology and pharmacology, we conducted studies to clarify the hypothetical direct or indirect interaction between sigma-1 receptors and N-type Ca^2+^ channels. To address the possible interaction, at first, we confirmed the expression of sigma-1 receptors in the cholinergic interneurons (ChIs) in rat striatum. Then we used the method of electrophysiology in brain slices to observe the influence on N-type Ca^2+^ channels by sigma-1 receptors in rat striatal ChIs. To evaluate whether the effect attributes to chaperone-mediated regulation induced by sigma-1 receptors, we co-expressed sigma-1 receptors and N-type Ca^2+^ channels in Xenopus oocytes. At last, we investigated the protein-protein interaction between sigma-1 receptors and N-type Ca^2+^ channels with techniques of FRET assays and Co-IP in HEK-293T cells.

## Materials and Methods

### Brain Slice Preparation and Solution

All experiments were performed in accordance with the NIH guideline to the care and use of laboratory animals (Publication NO.85-23, revised 1985) and approved by the Animal Research Advisory Committee of Beijing Institute of Biological Science. The procedures were similar to those we described previously (Zhao et al., 2016). In brief, male Sprague Dawley rats (14–16 days old) were decapitated. The brain was rapidly removed and submerged in oxygenated sucrose solution at 4°C containing (in mM): 2.5 KCl, 1.25 NaH_2_PO_4_, 24 NaHCO_3_, 10 glucose, 10 MgSO_4_, 0.5 CaCl_2_, 2 sodium pyruvate, 230 sucrose (295–305 mOsm/l). The value of pH was adjusted to 7.4 with NaOH. Coronal striatal slices (300 μm) were prepared with a vibratome (MA 752, Campden instruments). Slices were then transferred into a chamber (BSC-PC, Warner instruments) continuously bubbled with 95% O_2_ and 5% CO_2_ gas mixture for 30 min at 32°C. The solution incubating the slices was the standard NaHCO_3_-buffered saline solution containing (in mM): 126 NaCl, 2.5 KCl, 1.25 NaH_2_PO_4_, 26 NaHCO_3_, 10 glucose, 2 sodium pyruvate, 2 CaCl_2_, 2 MgCl_2_ (295–305 mOsm/l). pH was adjusted to 7.4 with HCl. The chamber was then maintained at room temperature bubbled with O_2_/CO_2_ gas mixture. Under this condition, the slices could be stored for several hours.

### Electrophysiology

Electrophysiological recording in the striatal ChIs was conducted as described previously (Zhao et al., 2016). Briefly, the pipettes had a resistance of 3–5 MΩ when filled with the internal solution consisted of (in mM): 80 CsOH, 80 gluconate acid, 30 CsCl, 40 HEPES, 10 tetraethylammonium chloride (TEA-Cl), 5 EGTA, 12 Na_2_phosphoceatine, 1 MgCl_2_, 2 Mg-ATP, 0.5 Na-GTP (265–270 mOsm/l), which was adjusted with CsOH to pH 7.3 (Miki et al., 2013). Slices were bathed in an external solution of (in mM): 105 NaCl, 20 TEA-Cl, 2 CaCl_2_, 6 MgCl_2_, 6 KCl, 26 NaHCO_3_, 10 glucose, 3 myo-inositol, 2 sodium pyruvate, 0.5 ascorbic acid, 1.25 NaH_2_PO4, 0.0005 tetrodotoxin (TTX, pH = 7.2 with TEA-OH, Miki et al., 2013). The slice in the recording chamber was visualized with a 40 × water-immersion objective (NIR Apo, Nikon, Japan) using standard infrared and differential interference contrast (IR-DIC) microscopy and a CCD camera. Cells in the striatum approximately 50 μm beneath the slice surface were patched. Electrophysiology was performed using an Axon 200B amplifier (Molecular devices, Foster city, CA, United States) and Clampex 10.1 software (Molecular devices) at room temperature 23 ∼ 25°C (Hawkins et al., 2015). Data were filtered at 2 kHz and digitized at 10 kHz online. Only those recordings with stable holding currents and access resistance were accepted.

### Immunohistochemical Staining

The rats of the same age to those used for electrophysiological experiments were used for histochemical experiments. The rats were anesthetized with Nembutal. After perfused with phosphate buffered saline (PBS) and fixed in 4% paraformaldehyde, the brain was removed carefully, cut sagittally at the midline and post-fixed in the same fixative for 3 h at 4°C (Zhao et al., 2016). The brain was sectioned at 5 μm to form the sequential brain slices. The slices containing the striatum were mounted on polylysine-coated slides. The staining was performed by using commercially available antibodies: a rabbit anti-sigma-1 receptor antibody (Abcam, Cat#ab53852, RRID: AB_881796 using at 1:200) and a goat anti-ChAT antibody (Millipore, Cat#AB144P, RRID: AB_2079751 using at 1:500).

Sections were washed with PBS for 3 × 5 min and blocked with normal serum consistent with the host of secondary antibody for 1 h at RT. After that, the serum was removed and primary antibody (diluted in PBS containing 1% BSA) was then applied for overnight at 4°C. Thereafter, sections were rinsed with PBS for 3 min × 5 min and stained with the secondary antibody in blocking serum for 1 h at room temperature in dark. At last, after another three washes, the sections were mounted in medium fluoroshield^TM^ with DAPI (Sigma–Aldrich F6057, Saint Louis, MO, United States) for visualization. Images were acquired using an Olympus microscope under 40× lens.

### Single-Cell Reverse Transcription-Polymerase Chain Reaction (scRT-PCR)

ScRT-PCR was performed using protocols previously described (Yan and Surmeier, 1996; Day et al., 2002; Zhao et al., 2016). Individual neuron was aspirated into micropipettes by applying negative pressure while being continuously perfused. After aspiration, the tip of the electrode was broken off and the content was expelled into a 0.5 ml Eppendorf (EP) tube containing 0.5 μl of random primer, 0.25 μl of RNasin (40 U/μl) and 4 μl of DEPC-treated water.

Single-stranded cDNA was acquired by reverse transcription. The neuron-containing mixture was heated to 70°C for 5 min to denature the nucleic acids, and then immediately chilled on ice for at least 5 min. The reverse transcription reaction mixture was added to the reaction tube. The reaction mixture contained primer mixture, GoScript^TM^ 5× Reaction buffer (4 μl), MgCl_2_ (2.4 μl), dNTP (1.0 μl), RNasin (0.25 μl), GoScript^TM^ Reverse Transcriptase (1.0 μl), nuclease-free water (6.35 μl), and was processed according to the following steps to synthesize the single-strand cDNA: annealing at 25°C for 5 min, extending at 42°C for 60 min, inactivating reverse transcriptase at 70°C for 15 min, and then icing. In the end, to eliminate any residual RNA, 1 μl of RNase H (2 U/μl) was added to the mixture that was incubated at 37°C for 20 min. All the reagents were from Promega Inc. (Madison, WI, United States).

The resulting cDNA from RT was amplified by using a modified protocol from Zhao’s report (Zhao et al., 2016). The amplification was carried out in a thermal cycler (Applied Biosystems). The PCR reaction mixture contained: 10 μl green GoTaq flexi buffer, 2.5 mM MgCl_2_, 0.2 mM each dNTP, 0.8 μM primers, 2 μl cDNA template, 125 U GoTaq G2 flexi DNA polymerase, and Nuclease-free water for a final volume of 50 μl. The thermal cycling protocol was set as: 94°C for 3min; and then 94°C for 1 min, 58°C for 1 min, 72°C for 1 min for 43 cycles; and followed by 72°C for 5 min.

The PCR primer sequences for β-actin, GAPDH, and ChAT are described in Yan and Surmeier (1996). The primer sequences of sigma-1 receptor and Ca^2+^ channels in scRT-PCR were reported by Zamanillo et al. (2000) and D’Ascenzo et al. (2004), respectively. The primers were synthesized by Beijing Biomed Inc. All the procedures were performed to minimize the cross-contamination (Mavournin et al., 1990). In each set of single-neuron reactions, negative controls were performed by omitting reverse transcriptase to ensure extraneous and genomic DNA did not contribute to the PCR product. To control for extraneous cDNA, the cellular template was replaced with Nuclease-free water. PCR products were then analyzed by the electrophoresis in 2% agarose gels with ethidium bromide staining. Both controls were consistently negative in these experiments.

### Molecular Biology (Fusion Proteins and Expression Vectors)

Full-length cDNA for human sigma-1 receptor variant 1 was obtained from OriGene (Rockville, MD, United States). The sequence is available under GenBank accession number NM_005866.2. For oocyte expression, sigma-1 receptor was subcloned into the pGH-19 vector modified by BamH I and EcoR I restriction enzymes sites. The orientation of sigma-1 receptor-pGH19 was T7 and the linearizing site for RNA was Xhol I. The Ca_v_2.2 (α1b) cDNA used in oocyte study was a gift from Dr. Diane Lipscombe (Lin et al., 1997). GenBank accession number of the α1b, β1b, and α2δ1 cDNA sequence is AF055477, L06110, and AF296488, respectively. The sigma-1 receptor cDNA was also subcloned into the CMV promotor-driven eukaryotic expression vector Dsred-N1 (Clontech) for expression in HEK-293T cells. The construct encoding the full-length GFP-tagged Ca_v_2.2 subunit was a generous gift from Dr. Gerald W. Zamponi (Kisilevsky et al., 2008).

### Oocyte Expression and Electrophysiology

Oocytes were harvested from anesthetized Xenopus laevis through a small abdominal incision. The follicular membranes were removed by digesting in OR2 solution (82.0 mM NaCl, 2.5 mM KCL, 1.0 mM MgCl_2_, 5.0 mM HEPES, pH 7.6 adjusted with NaOH) containing 1.5 mg/ml collagenase type I (Sigma–Aldrich, St. Louis, MO, United States) for total of 1 h at room temperature. Oocytes were then incubated in ND-96 solution (96.0 mM NaCl, 2.0 mM KCl, 1.0 mM MgCl_2_, 1.8 mM CaCl_2_, 5.0 mM HEPES, and 2.0 mM sodium pyruvate. pH was adjusted to 7.4 with NaOH) at 18°C for at least 12 h before injection. With a microinjection pipette, 46 nl cRNA was injected into oocytes. The injection was carried out using Drummond nano-injector (Drummond Scientific Co., Broomall, PA, United States). Oocytes were then cultured for 48–96 h before recording. cRNA was synthesized from a T7 promotor using the Ribo MAX^TM^ Large Scale RNA Production System (Promega, Gaithersburg, MD, United States), which was diluted in nuclease-free water to different concentrations.

After 48 h, currents were recorded using an Axon 2B amplifier (Molecular devices, Foster city, CA, United States) and Clampex 10.1 software (Molecular devices) at room temperature. We recorded the N-type Ca^2+^ current using the following voltage protocol: the holding potential was held at -80 mV and the testing potential was stepped from -80 to +50 mV for 500 ms with an increment of 10 mV. Oocytes were impaled with two electrodes pulled from borosilicate glass pipettes and filled with 3 M KCl. Electrode resistance was 0.5–2.5 MΩ. Recording was performed with oocytes bathing in the external solution containing (in mM): 5 BaCl_2_, 50 *N*-methyl-d-glucamine, 5 KCl, 5 HEPES, and pH was adjusted with methanesulfonic acid to 7.4 (Zhang et al., 2015). Leak and capacitive currents were subtracted using the P/4 procedure.

### Cell Culture and Transient Expression

HEK-293T cells were cultured in Dulecco’s modified Eagle’s medium (DMEM, Invitrogen) containing10% fetal bovine serum (v/v), 50 U/ml penicillin, and 50 μg/ml streptomycin at 37°C supplied with 5% CO_2_. Poly-l-lysine coated coverslips were put at the bottom of 35 mm dish. The cells were planted on the coverslips for transfection. The cells were then transfected with the transfection reagent PolyJet (SignaGen) in accordance with the manufacture’s protocol at a 3:1 reagent to DNA ratio (Cassidy et al., 2014). The DNA mixtures (1 μg) used in the transfection consisted of pEGFP-N1-Cacna1b, pcDNA3.1_Cacnb1b, and pcDNA3.1_Cacna2d1 at a ratio of 2:1:1. The medium was replaced 16 h after transfection. Forty-eight hours after transfection, the cells were washed three times in PBS for 5 min each time and mounted them with DAPI. Then fluorescence was observed using confocal microscopy (Olympus).

### Fluorescence Resonance Energy Transfer (FRET) Assay

FRET was measured in HEK-293T cells that were co-transfected with sigma-1 receptor-Dsred and pEGFP-Ca_v_2.2 (α1b + β1b + α2δ1) using PerkinElmer Ultra IEWVox. The pEGFP-Ca_v_2.2 was the donor and sigma-1 receptor-Dsred was the acceptor. Band limited excitation (420–495 nm) was provided by a laser light and three filters. Cells were imaged using an inverted microscope (Zeiss LSM-880) and an oil-immersion 40× objective lens. Data analysis was done with the Volocity 6.0. The method of nFRET was as shown in Xia and Liu (2001). The equation was as following: NFRET = (FRET-(Acceptor × A)-(Donor × B))/sqrt (Acceptor × Donor).

### Co-immunoprecipitation

All processes described below were performed on ice except noted specifically. Sigma-1 receptor-Dsred and pEGFP-Ca_v_2.2 expressing cells were washed three times with cold PBS, then lysed with 500 μl RIPA Lysis buffer (CW Biotech) and incubated on ice for 30 min. Lysates were centrifuged at 12000 *g* for 20 min at 4°C, and the supernatants were removed to another EP tube. Protein concentration of cellular extracts was measured using a BCA assay kit (Thermo Scientific). After that, 500 μg of supernatants were mixed with 1 μg of the homolog antibody: the anti-sigma-1 receptor antibody (Abcam, Cat#ab53852, RRID: AB_881796), the anti-Ca_v_2.2 antibody (Millipore, Cat#AB5154, RRID: AB_2069093), or 1 μg IgG (Sigma–Aldrich, Cat#I8140, RRID: AB_1163661), and rotated at 4°C for 4 h. Then, 20 μg agrose A beads (Vigorous Biotechnology) were added to each sample that was rotated continuously at 4°C for overnight. Thereafter, samples were centrifuged at 3000 rpm for 5 min at 4°C, and then the supernatants were removed. Immunoprecipitants were washed twice with 1 ml of 1× lysis buffer, then once with 1 ml of PBS for 5 min at 3000 rpm each time. Both washing buffers contained 10 μl of protease inhibitor. After wash, samples were boiled in 60 μl of 2 × SDS loading buffer at 95°C for 10 min. Proteins were probed with anti-sigma-1 receptor antibody (Proteintech, Cat#15168-1-AP, RRID: AB_2301712 using at 1:500) and anti-Ca_v_2.2 antibody (Proteintech, Cat#19681-1-AP, RRID: AB_10638918 using at 1:500) separately. Bands were visualized with an ECL (Gene Co. Ltd) (Kourrich et al., 2013; Yan et al., 2014).

### Chemicals and Data Analysis

Chemicals were obtained from Sigma–Aldrich (St. Louis, MO, United States), TCI (Shanghai, China), TOCRIS bioscience (Bristol, United Kingdom). SKF-10047, BD-1063, PRE-084, and NE-100 were dissolved in nuclease-free water to form a 10 mM stock solution and further diluted in the bathing solution for the final concentration that were perfused at a rate of 2 ml/min by a peristaltic pump. Data analysis was performed with softwares including Clampfit Version 10.2, Prism Version 6.0 and Origin Version 9.0. The significance of the fit parameters (Mean ± SEM) was tested using student’s *t*-test, one-way ANOVA, and two-way ANOVA. Difference of *p* < 0.05 was considered statistically significant.

## Results

### N-type Ca^2+^ Channels and Sigma-1 Receptors Expression on ChIs

To observe a possible interaction between sigma-1 receptors and N-type Ca^2+^ channels, we had to use a specific cell that expressed both of these proteins. It was reported that N-type Ca^2+^ channel was the dominant one among all the voltage-gated Ca^2+^ channels expressed in ChIs (Yan and Surmeier, 1996). However, the expression of sigma-1 receptor in ChIs is not reported until now. Therefore, the expression of sigma-1 receptors and N-type Ca^2+^ channels in ChIs should be clarified firstly.

**FIGURE 1:**
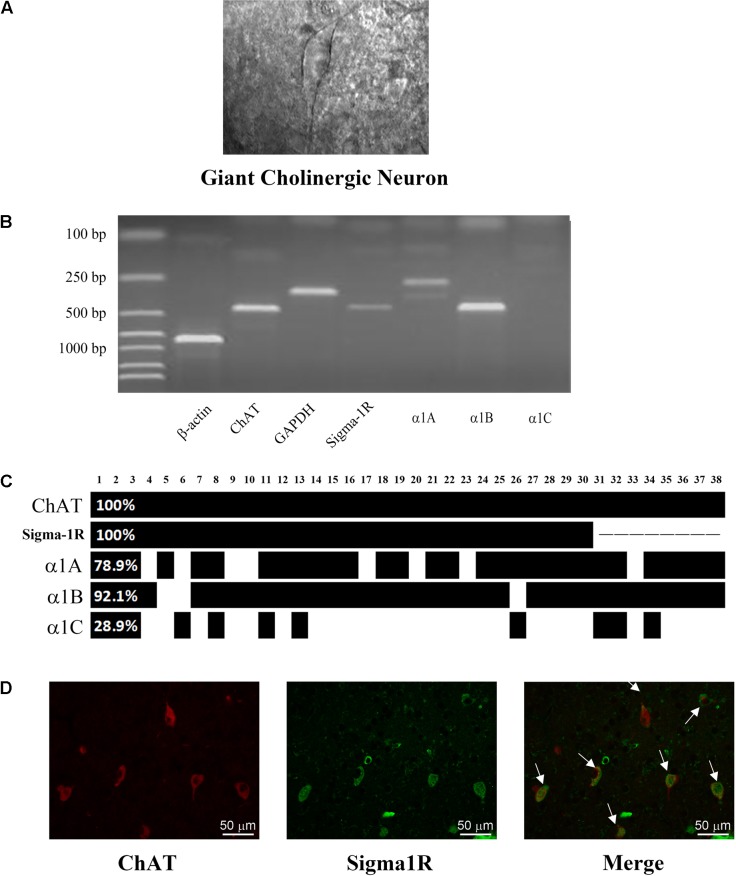
**Identification of the expression of N-type Ca^2+^ channel and sigma-1 receptor in ChIs. (A)** An IR-DIC image of a striatal slice illustrating the characteristic appearance of giant cholinergic interneurons (ChIs). **(B)** Representative photograph of single-cell reverse transcription polymerase chain reaction (scRT-PCR). Experiment products were stained in a 2% agarose gel. Molecular mass markers were shown in the first lane. β-actin and GAPDH were introduced as positive control. The presence of ChAT indicated the aspirated cell was a ChI. **(C)** Bar plot indicated the co-expression of ChAT, sigma-1 receptor and α1A-C in ChIs by scRT-PCR. ChAT and sigma-1 receptor were in every detected neuron. The α1B subunit of N-type Ca^2+^ channel was found in 92.1% detected cells. **(D)** Immunofluorescence of successive paraffin-embedded rat striatum slice observed under 40× objective lens. Left: using the anti-sigma-1 receptor antibody at a dilution of 1:20 and FITC-conjugated affinipure goat anti-rabbit IgG. Middle: Using the anti-ChAT antibody at a dilution of 1:200 and TRITC-conjugated affinipure rabbit anti-goat IgG. Right: the merged picture of the sigma-1 receptor and ChAT.

Cholinergic interneurons were picked out by their large and specific shape in rat striatal slices (**Figure 1A**). scRT-PCR was performed by sucking the cellular content without nucleus into the recording pipette. PCR products were separated by the electrophoreses in 2% agarose gels, stained with ethidium bromide and visualized by UV light. In **Figure 1B**, the positive ChAT lane revealed that the tested cell was ChI neuron, and sigma-1 receptors, Ca_v_α1A and α1B were observed in the same neuron. For analysis of calcium channel expression, the same experiment was performed on 38 ChIs and the summarized result was plotted in **Figure 1C**. Briefly, three types of voltage-gated Ca^2+^ channels were found with different detection rates of 78.9% (α1A, P/Q-type), 92.1% (α1B, N-type), and 28.9% (α1C, L-type), respectively. It was obvious that N-type Ca^2+^ channel was the prominent isoform in all the high-voltage gated Ca^2+^ channels in ChIs.

Meanwhile, we detected the expression of sigma-1 receptor on 30 neurons which are included in the 38 neurons. The mRNA of sigma-1 receptor was found in every tested ChI neuron (**Figure 1C**). There were about 90% (27/30) ChIs that co-expressed the sigma-1 receptors and N-type Ca^2+^ channels. The protein of sigma-1 receptor in ChIs was further verified by using immunofluorescence staining on sequential brain slices (5 μm). Two adjacent slices were separately incubated with goat anti-ChAT and rabbit anti-Sigma-1 receptor antibodies. Red immunofluorescence of ChAT staining, as illustrated in **Figure 1D**, showed the morphology and the localization of ChIs, while the green staining represented sigma-1 receptor. When two slices were merged, colocalization of both ChAT and sigma-1 receptor was clearly observed in several ChIs (as indicated by the arrows). In combination with scRT-PCR, these results proved that sigma-1 receptors were also expressed in ChIs.

### Inhibition of Ca^2+^ Current by Sigma-1 Receptor Agonist in Rat Striatum ChIs

**FIGURE 2:**
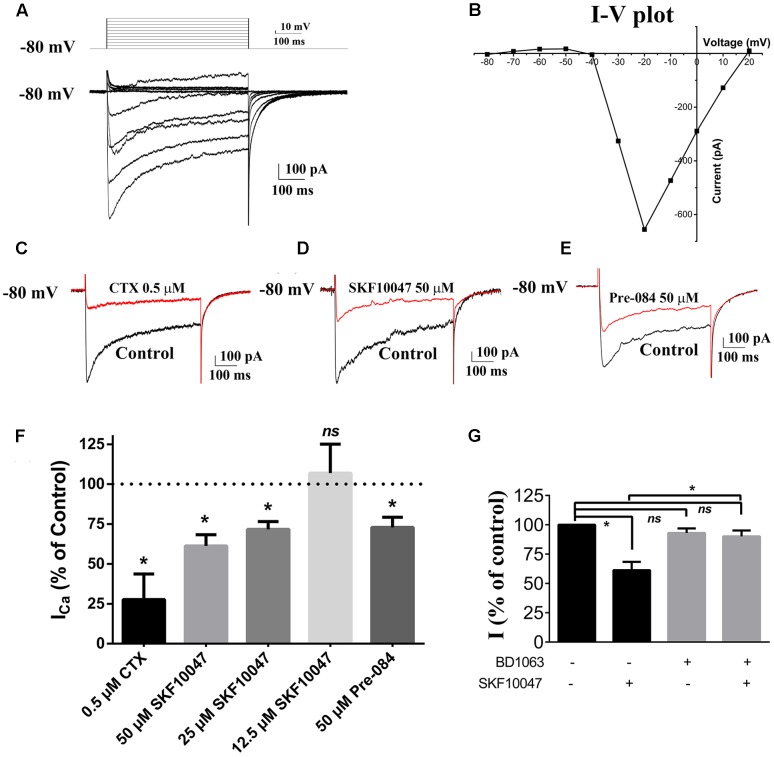
**Sigma-1 receptor agonist reduced currents of Ca^2+^ channel in ChIs. (A)** Representative trace of Ca^2+^ channel current obtained from the rat striatum ChIs in the brain slice. **(B)** I–V plot of the recorded Ca^2+^ channel current trace in **(A)**. **(C–E)** Representative traces of current evoked before and after the bath application of 0.5 μM CTX, 50 μM SKF-10047, and 50 μM Pre-084, respectively. **(F)** Summarized bar graph to present the effect of CTX (N-type Ca^2+^ channel blocker), different concentrations of SKF-10047 and Pre-084 (sigma-1 receptor agonists) on Ca^2+^ currents. **(G)** Summarized bar graph to present the effect of BD-1063 (sigma-1 receptor antagonist) on the Ca^2+^ current inhibition induced by SKF-10047. ^∗^*p* < 0.05.

To investigate whether the sigma-1 receptor can regulate the N-type Ca^2+^ channel, the Ca^2+^ current of ChIs in the striatal slice was first observed using the whole-cell recording technology. Ca^2+^ currents were evoked by a stimulating protocol as following: the holding potential was -80 mV and the testing potential was stepped from -80 mV to +20 mV for 500 ms with an increment of 10 mV. Recorded Ca^2+^ current was shown in **Figure 2A** and its related I–V curve was plotted in **Figure 2B**. When a specific N-type Ca^2+^ channel blocker, ω–conotoxin-GVIA (CTX, 0.5 μM), was applied, the current was inhibited by 72.3% (*n* = 3, *p* < 0.05; **Figures 2C,F**), which further confirmed that N-type Ca^2+^ channels were predominant voltage-gated Ca^2+^ channels in rat striatal ChIs.

The modulation of sigma-1 receptors on N-type Ca^2+^ channels was observed by using sigma-1 receptor agonists of SKF-10047 and Pre-084. When SKF-10047 was added to the bath solution, the amplitude of Ca^2+^ current was inhibited by 28.4% ± 4.9% (*n* = 9, *p* < 0.05) and 38.7% ± 7.1% (*n* = 11, *p* < 0.05) at the concentration of 25 and 50 μM, respectively (**Figures 2D,F**). Pre-084 (50 μM) also depressed the currents by 27.1% ± 6.4% (*n* = 7, *p* < 0.05) (**Figures 2E,F**). BD-1063, a selective antagonist of sigma-1 receptor, did not display any visible effect on Ca^2+^ currents. However, the inhibition of SKF-10047 on Ca^2+^ currents was abolished in the presence of BD-1063 (**Figure 2G**).

### Direct Interaction between Sigma-1 Receptors and N-type Ca^2+^ Channels

**FIGURE 3:**
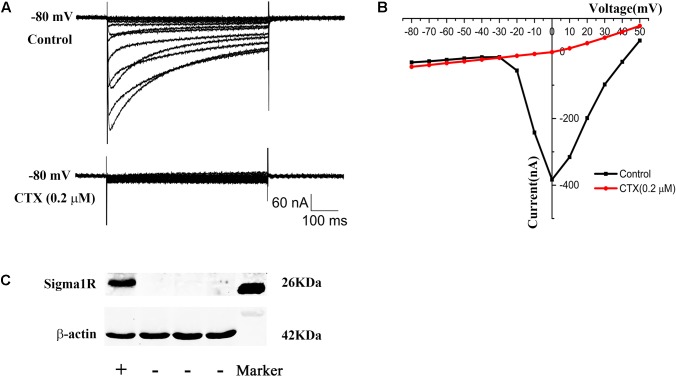
**Expression of N-type Ca^2+^ channel and sigma-1 receptor in xenopus oocytes. (A)** Upper panel: the current of N-type Ca^2+^ channel recorded in oocytes after microinjection with N-type Ca^2+^ channel cRNA. Lower panel: the current was blocked by N-type Ca^2+^ channel blocker CTX (0.2 μM). **(B)** I–V plot of the recorded N-type Ca^2+^ channel current in **(A)**. **(C)** Schematic Western blot strap of oocytes injected with sigma-1 receptor cRNA (the first lane) and control group (the middle three lanes), which indicated the sigma-1 receptor expressed successfully. The last lane showed the Marker.

In order to investigate the possible constitutive interaction between sigma-1 receptors and N-type Ca^2+^ channels, the two proteins were co-expressed in Xenopus oocytes. N-type Ca^2+^ channel cRNA contained α1b, β1b, and α2δ1 in a 2:1:1 ratio. No visible currents were evoked in native oocytes. However, big inward currents were recorded after cRNA of N-type Ca^2+^ channel was injected into Xenopus oocytes after 48 h and CTX (0.2 μM) blocked the currents completely (**Figures 3A,B**), which indicated that functional N-type Ca^2+^ channels were successfully expressed in oocytes. After sigma-1 receptor cRNA was injected into oocytes for 48 h, Western Blot was performed. The schematic Western blot strap in the left lane showed the sigma-1 receptor expressed successfully in oocytes (**Figure 3C**).

**FIGURE 4:**
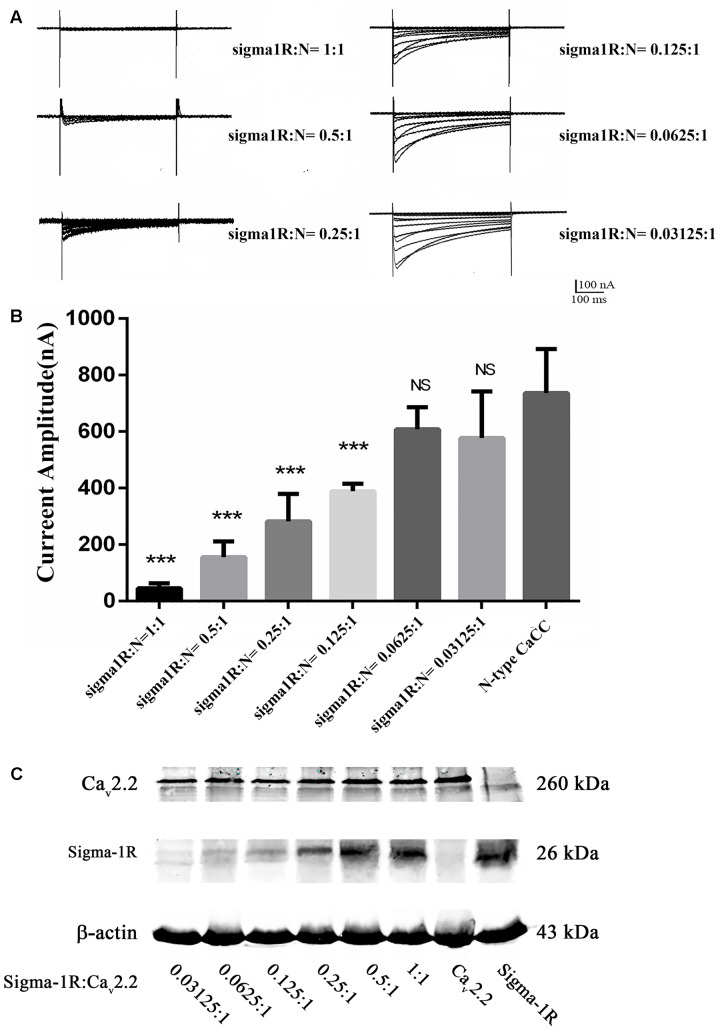
**Currents recorded at different cRNA ratio of sigma-1 receptor to N-type Ca^2+^ channel. (A)** Currents recorded from the oocytes injected with different cRNA ratio. The cRNA concentration ratio of Sigma-1 receptor: N-type Ca^2+^ channel = 1:1, 0.5:1, 0.25:1, 0.125:1, 0.0625:1, and 0.03125:1, respectively. **(B)** Summarized bar graph to present the N-type Ca^2+^ currents decreased with the increase of sigma-1 receptor cRNA injected in oocytes. The current amplitude recorded from oocytes with injection of N-type Ca^2+^ channel cRNA only was normalized as 100%. Values are Mean ± SEM (*n* = 18 for 1:1, *n* = 18 for 0.5:1, *n* = 17 for 0.25:1, *n* = 26 for 0.125:1, *n* = 17 for 0.0625:1, and *n* = 10 for 0.03125:1 group, respectively. ^∗∗∗^*p* < 0.001, ^∗^*p* < 0.05). **(C)** The expression levels of the two proteins from oocytes injected with the cRNA ratio of sigma-1 receptor: N-type Ca^2+^ channel at 0.03125:1, 0.0625:1, 0.125:1, 0.25:1, 0.5:1, and 1:1 from left to right. The blots in the right two lanes showed the protein expression in oocytes of control groups, which were injected with the N-type Ca^2+^ channel cRNA or the sigma-1 receptor cRNA alone.

To observe the possible interaction between sigma-1 receptors and N-type Ca^2+^ channels, a mixture of their cRNA was injected into oocytes with a ratio of 1:1. Surprisingly, no visible currents were induced by a depolarized stimulation. To clarify a possible chaperone-mediated interaction between sigma-1 receptors and N-type Ca^2+^ channels, we decreased the concentration of sigma-1 receptor cRNA and mix it with the constant concentration of N-type Ca^2+^ channel cRNA at the ratio of 0.5:1, 0.25:1, 0.125:1, 0.0625:1, and 0.03125:1, respectively. The results showed that the amplitude of N-type Ca^2+^ channel current was increased obviously with decreased sigma-1 receptor cRNA (**Figures 4A,B**). Compared to the currents recorded in oocytes with only expression of N-type Ca^2+^ channels, the current amplitudes were 29.9% ± 3.9% (*n* = 18, *p* < 0.001), 52.6% ± 3.8% (*n* = 17, *p* < 0.001), 72.1% ± 3.7% (*n* = 26, *p* < 0.001), 85.0% ± 5.8% (*n* = 17, *p* < 0.05), and 89.9% ± 7.5% (*n* = 10, *p* > 0.05) with the cRNA ratio of 0.5:1, 0.25:1, 0.125:1, 0.0625:1, and 0.03125:1, respectively. However, the expression levels of the two proteins was positively correlated with the amount of cRNA injected, indicating that the expression of each protein was not affected by each other (**Figure 4C**). These results suggested that sigma-1 receptors probably regulated N-type Ca^2+^ channels constitutively by direct interaction. As a negative control, we studied sigma-1 receptor and another ion channel, HCN1, in the same way as with N-type Ca^2+^ channel in oocytes. We found HCN1 current was not affected by sigma-1 receptor. This experiment indicated us the effect on N-type Ca^2+^ channels was specific (Data was not shown).

### Effect of Sigma-1 Receptor Agonist on N-type Ca^2+^ Channels

**FIGURE 5:**
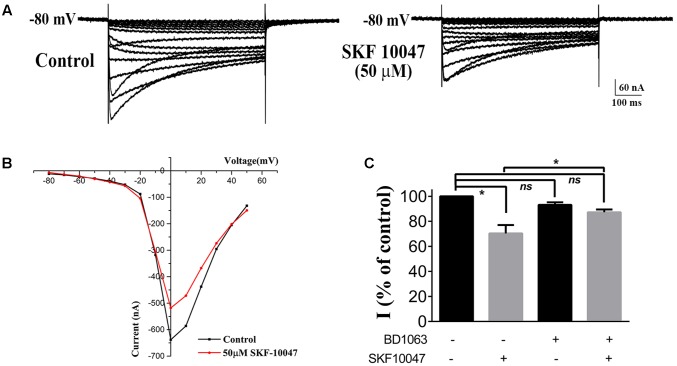
**Effect of sigma-1 receptor agonist and antagonist on N-type Ca^2+^ channel. (A)** Example trace of the inhibition on N-type Ca^2+^ current induced by 50 μM SKF-10047. The oocyte was injected with a cRNA ratio of sigma 1R: N-type Ca^2+^ channel = 0.25:1. **(B)** I–V plot of the recorded N-type Ca^2+^ current from the oocyte in **(A)**. **(C)** Summarized bar graph to present the effect of SFK-10047 (sigma-1 receptor agonist) and BD-1063 (sigma-1 receptor antagonist) on N-type Ca^2+^ currents. Two-way ANOVA analysis, Mean ± SEM, ^∗^*p* < 0.05.

To prove if sigma-1 receptor agonists could modulate the Ca^2+^ channel in oocytes the same way as in ChIs, we observed the effect of SKF-10047 on the currents recorded in oocytes in which sigma-1 receptor to N-type Ca^2+^ channel was injected at 0.25:1 ratio (**Figure 5A**). When SKF-10047 (50 μM) was added into the bath solution, the current was inhibited by 29.7% ± 6.7% comparing with control (**Figures 5A–C**, *n* = 6, *p* < 0.05). BD-1063 (50 μM) alone did not present remarkable inhibition on N-type Ca^2+^ currents (93.2 ± 2.1% of control, **Figure 5C**, *n* = 6, *p* > 0.05), but could block the inhibition of SFK-10047 on N-type Ca^2+^ current (87.2 ± 2.2% of control, **Figure 5C**, *n* = 6, *p* > 0.05). This result indicated that SKF-10047 inhibited N-type Ca^2+^ channel through sigma-1 receptor activation.

### Expression of Sigma-1 Receptors and N-type Ca^2+^ Channels in HEK-293T Cells

**FIGURE 6:**
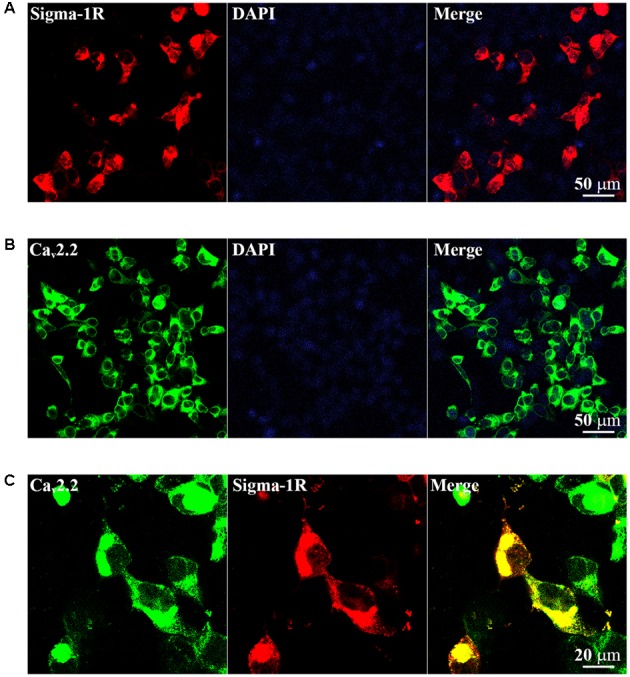
**Exogenous expression of Sigma-1-Dsred receptor and Ca_v_2.2-GFP in HEK-293T cell line. (A)** Fluorescence images of HEK-293T cells transfected with 0.5 μg Sigma-1-Dsred receptor cDNA. **(B)** Fluorescence images of HEK-293T cells transfected with 0.5 μg Ca_v_2.2-GFP (α1b+β1b+α2δ1subunits) cDNA. **(C)** Confocal microscopy images of HEK-293T cells with both 0.5 μg Ca_v_2.2-GFP and 0.5 μg Sigma-1-Dsred receptor cDNA. Co-localization was shown in yellow.

As reviewed in Su et al. (2016), we speculated that the inhibition of sigma-1 receptor on N-type Ca^2+^ channels may be mediated by protein–protein interaction. To investigate if there is a protein–protein interaction between receptor and channel, we took HEK-293T cells as an exogenous expressing model. Cultured HEK-293T cells were transiently transfected with the GFP-Ca_v_2.2 channels (α1b+β1b+α2δ1subunits) in the absence and presence of sigma-1-Dsred receptor. The cells were observed using confocal microscopy. As illustrated in **Figures 6A,B**, the red and green fluorescence indicated sigma-1 receptors and N-type Ca^2+^ channels were successfully expressed, respectively. When cells were co-transfected with sigma-1 receptor and N-type Ca^2+^ channel, there is yellow fluorescence in merged channel. This indicated that receptors and channels were co-expressed in the same cell, as shown in **Figure 6C**. These results allowed us to further study the interaction of the receptors and channels.

### Sigma-1 Receptor Physically Associates with Ca_v_2.2 Channels in HEK-293T Cells

**FIGURE 7:**
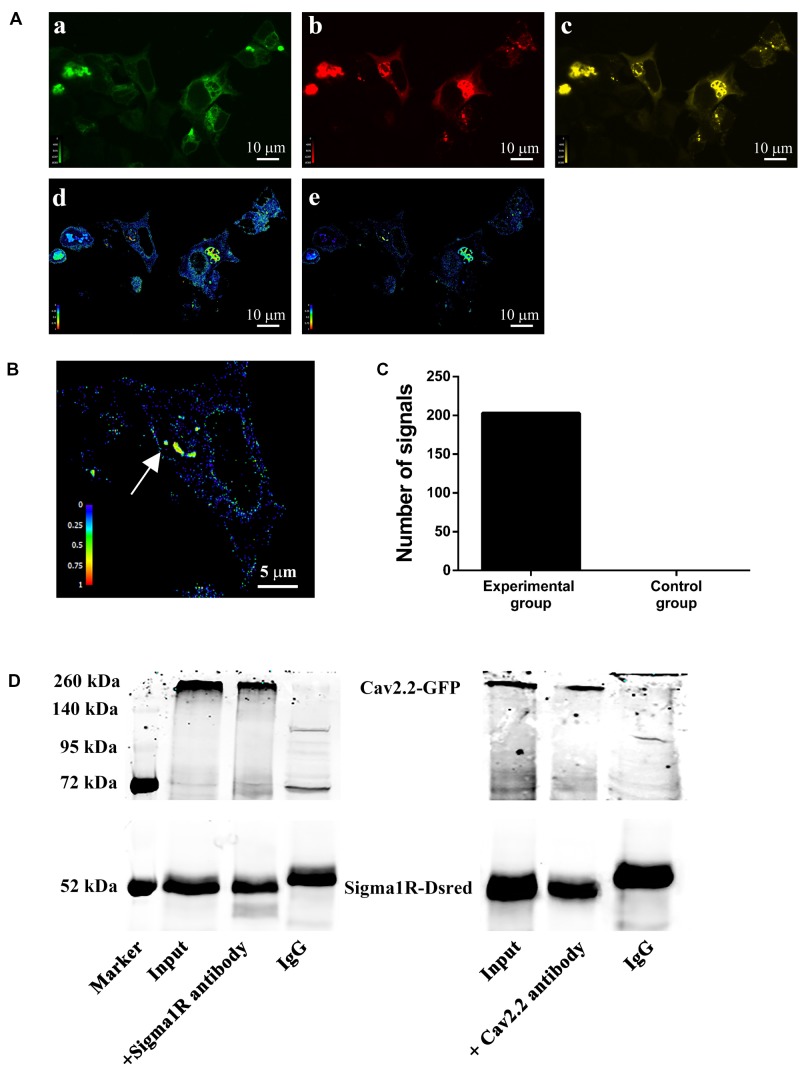
**Protein–protein interaction between sigma-1 receptors and N-type Ca^2+^ channels. (A)** HEK-293T cells were transfected with both Ca_v_2.2-GFP (α1b+β1b+α2δ1) and sigma-1-Dsred receptor. Pseudo-color images were obtained under the three filter sets: GFP (a), Dsred (b), and FRET (c). After subtraction of background and bleed-through signals, net FRET (d) was acquired. Normalized FRET (e) values were got by using equation described in section “Materials and Methods.” Color bars represented relative degree of net FRET and normalized FRET within the cells. **(B)** Representative cell enlarged from (e). The net FRET value of the point with the arrow was 0.81 ± 0.06 (Mean ± SD). **(C)** Bar graph of the statistical numbers in 11 experimental fields (203) and 5 negative control fields (0). **(D)** Co-immunoprecipitation of GFP-Ca_v_2.2 channels and sigma-1-Dsred receptors. Total lysates were prepared from HEK-293T cells. Immunoprecipitated samples were run on the gels and the blots probed with either anti-Ca_v_2.2 antibody or anti-sigma-1 receptor antibody.

To examine a direct interaction between sigma-1 receptors and N-type Ca^2+^ channels, the technology of FRET was performed. Using the co-transfected HEK-293T cells, we chose GFP-Ca_v_2.2 as the FRET donor and sigma-1 receptor-Dsred as the acceptor. In the experimental group, we used 488 nm laser-light to stimulate the donor (GFP), which then excites the red fluorescence from FRET channel when both are in close proximity as shown in **Figure 7A**. Using the Volocity 6.0, we measured the intensity of the signals from nFRET channel. We set the area of signals 0.02 μm^2^ and the intensity ≥0.5 as valid standards. Then we counted the number of signals under this condition. In experimental group, we obtained 203 signals from 11 fields subjected to the condition (**Figure 7C**). An example including the signals in this field was shown in **Figure 7B**, where the nFRET value of each signal was 0.81 ± 0.06 (Mean ± SD). However, in the control experiment, where pEGFP vector alone was co-transfected with sigma-1 receptor-Dsred, the number of signals matched the condition was 0 (zero) (also see **Figure 7C**). This indicated the existence of the receptor-channel complex.

To investigate the receptor-channel physical interaction further, we performed the co-immunoprecipitation. Samples were processed with anti-Ca_v_2.2 antibody or anti-sigma-1 receptor antibody. Probing the gel with antibody against N-type Ca^2+^ channel in the sample immunoprecipitated with anti-sigma-1 antibody revealed a band at 260 kDa shown in **Figure 7D** left panel. On the other hand, probing with antibodies against sigma-1 receptor in the sample immunoprecipitated with anti-N-type Ca^2+^ channel antibody revealed a band at 52 kDa, as shown in **Figure 7D** right panel. The input of the cell lysates indicated that the receptor and the channel were co-expressed, IgG was used as negative control, as shown in **Figure 7D**. These results demonstrated the physical association of sigma-1 receptors with N-type Ca^2+^ channels.

## Discussion

Since the sigma-1 receptor was discovered decades ago (Martin et al., 1976; Su, 1982), it has been investigated broadly in many aspects. It is now recognized as an ligand-regulated chaperone protein that resides at MAM and some plasma membrane compartments (Hayashi and Su, 2007). The human crystal structure of the sigma-1 receptor reveals a trimeric organization and each promoter contains only a single transmembrane domain, which includes a cupin-like β-barrel with the ligand-binding site buried at its center (Schmidt et al., 2016). The unique structural features of sigma-1 receptors probably underwrite its multitasking functions. The receptor has been demonstrated to be involved in various biological and pathological processes, and has been considered as an important therapeutic target in many central nervous system diseases in human (Maurice and Lockhart, 1997; Maurice et al., 2002; Maurice and Su, 2009). The N-type Ca^2+^ channel is well established as a critical factor to control neurotransmitter release. The N-type Ca^2+^ channel mainly locates at presynaptic membrane and mediate rapid Ca^2+^ influx into the synaptic terminal that triggers synaptic vesicle exocytosis and neurotransmitter release (Llinás et al., 1981; Catterall, 2000; Momiyama and Koga, 2001). Accumulative evidence reveals that sigma-1 receptors are also involved in the regulation of neurotransmitter release. Sigma-1 receptor activation inhibits glutamate release from rat cortical nerve terminals, which is prevented by blocking N-type and P/Q-type Ca^2+^ channels, but not by blocking the ryanodine receptors or the mitochondrial Na^+^/Ca^2+^ exchange (Lu et al., 2012). In rat primary retinal ganglion cells (RGCs), sigma-1 receptor agonist inhibits calcium currents and an association between L-type calcium channels and the sigma-1 receptors is demonstrated (Tchedre et al., 2008). Despite many kinds of VGCCs appear to be constitutively regulated by direct interaction with sigma-1 receptors (Chu and Ruoho, 2015), it still remains unclear that if there is an interaction between sigma-1 receptors and N-type Ca^2+^ channels.

To identify the possible interaction between sigma-1 receptors and N-type Ca^2+^ channels, we need to find a specific cell that has both proteins co-expressed. ChIs in rat striatum has been reported to express several subtypes of VGCCs, and the N-type Ca^2+^ channel is predominant one (Yan and Surmeier, 1996). However, it is unclear if sigma-1 receptors are also expressed in ChIs. In the experiment, we first identify that sigma-1 receptors are expressed in ChIs by using scRT-PCR and immunofluorescence staining techniques. Three subtypes of VGCCs (N-type, P/Q-type, and L-type) present a different detection rate and N-type Ca^2+^ channels are found nearly in every ChI. A large portion (72.3%) of VGCC currents induced in ChIs is inhibited by CTX, a specific N-type Ca^2+^ channel blocker, which further confirms that N-type Ca^2+^ channels are predominant voltage-gated Ca^2+^ channels, and the result is identical with Yan’s reports (Yan and Surmeier, 1996). When agonists of sigma-1 receptors (SKF-10047, Pre-084) are applied into the bath solution, the VGCC currents are depressed remarkably. The sigma-1 receptor antagonist (BD-1063) alone is not able to block the Ca^2+^ currents, but it completely abolishes the blockage induced by sigma-1 receptor agonist SKF-10047, which suggests the receptor activation underlies the mechanism of the inhibition. Agonist of sigma-1 receptors has also been reported to block VGCC currents in neurons and other tissues and it is considered as a directly activation of the receptors at the level of the plasma lemma (Zhang and Cuevas, 2002; Tchedre et al., 2008; Lu et al., 2012; Pan et al., 2014). However, the evidence for a direct interaction between sigma-1 receptors and N-type VGCC is lacking.

To observe the possible interaction between sigma-1 receptors and N-type Ca^2+^ channels, Xenopus laevis oocyte is used to co-express both of the two proteins. The results show that no currents are evoked when cRNAs of sigma-1 receptors and N-type Ca^2+^ channels are injected into oocytes at a concentration ratio of 1:1. The possible explanation may be a chaperon-mediated interaction induced by sigma-1 receptors. To verify the consideration, we reduce the concentration of sigma-1 receptor cRNA and mix it with a fixed concentration of N-type Ca^2+^ channel cRNA. Then N-type Ca^2+^ channel currents appear and their amplitudes increase with decreased sigma-1 receptor cRNA. In this situation, sigma-1 receptor agonist can still display its blockage on N-type Ca^2+^ currents. Even though the relationship between ligand binding to sigma-1 receptor and the subsequent biological response is not well addressed, evidence for ligand-mediated changes in sigma-1 receptor oligomerization state is confirmed (Mishra et al., 2014). Sigma-1 receptor agonists alter oligomeric structures and favor dissociation of the complexes of the receptor. Therefore, we consider that sigma-1 receptors inhibit N-type Ca^2+^ channels probably by two mechanisms: a direct physical interaction constitutively and a conformational change induced by receptor activation at the surface of cell membrane. To clarify the molecular basis of interactions between sigma-1 receptors and N-type Ca^2+^ channels, HEK-293T cells are used to express both proteins. The fluorescence indicates that sigma-1 receptors and N-type Ca^2+^ channels are successfully co-expressed in HEK-293T cells. FRET experiments reveal that sigma-1 receptors and N-type Ca^2+^ channels locate in a close proximity. However, the three-filter system is affected by a number of factors, e.g., photobleaching/quenching, quantum yield of fluorophores, and molecular position/orientation, and critical in collecting data (Xia and Liu, 2001). To derive energy transfer efficiencies accurately, we should perform the experiments combining the three-filter system with acceptor photobleaching. Co-IP experiment displays a protein complex formed by sigma-1 receptors and N-type Ca^2+^ channels. These results establish the molecular basis for the protein–protein interaction between sigma-1 receptors and N-type Ca^2+^ channels.

The sigma-1 receptor agonists have recently attracted much attention as potential therapeutic drugs for cognitive and affective disorders. However, it is still unclear whether they act via modulation of neurotransmitter release (Antonini et al., 2011). As our data reveal that agonists of sigma-1 receptor block N-type Ca^2+^ channels, neurotransmitter release is probably also inhibited while the agonists are administrated. The negative modulation of sigma-1 receptor and its agonists on N-type Ca^2+^ channels might partially contribute to their therapeutic effect on nervous system disorders.

## Conclusion

Our data first identify that the sigma-1 receptor is expressed in rat striatal ChIs and agonists of sigma-1 receptors can depress VGCC currents by activating the receptors. Heterogeneous express experiment demonstrates that sigma-1 receptors and N-type Ca^2+^ channels form a complex on the plasma lemma by Co-IP and FRET assays. Via the protein-protein interaction, sigma-1 receptors exercise negative modulation on N-type Ca^2+^ channels by a direct physical contact and an agonist-induced conformational change. Considering the critical regulation of N-type Ca^2+^ channels on neurotransmission, our data should be benefit to better understand the multitasking functions of sigma-1 receptors.

## Author Contributions

Study concept design: KZ, ZZ, and JZ. Collection of data: KZ and ZZ. Analysis and interpretation of data: KZ, ZZ, and JZ. Drafting of the manuscript: KZ and ZZ. Critical revision of the manuscript: KZ and JZ. Study supervision and help: LL, XL, XW, LW, and HY. All authors approved the final version of the manuscript. All experiments were performed in State Key Laboratory of Toxicology and Medical Countermeasures in China.

## Conflict of Interest Statement

The authors declare that the research was conducted in the absence of any commercial or financial relationships that could be construed as a potential conflict of interest.
